# Predicting the future for neonates with symptomatic congenital heart disease

**DOI:** 10.1038/s41372-026-02653-6

**Published:** 2026-04-13

**Authors:** Mahati Pidaparti, Benjamin M. Helm, Britney Reed, Paulomi M. Chaudhry, Gabrielle Geddes, Melinda H. Markham, Poonam M. Puranik, Kristen R. Suhrie

**Affiliations:** 1 Indiana University School of Medicine, Department of Pediatrics, Division of Neonatal-Perinatal Medicine, Indianapolis, IN USA; 2 Indiana University School of Medicine, Department of Medical and Molecular Genetics, Indianapolis, IN USA; 3 Indiana University School of Medicine, Indianapolis, IN USA; 4 Indiana University School of Medicine, Department of Pediatrics, Division of Cardiology, Indianapolis, IN USA

**Keywords:** Paediatrics, Outcomes research

## Abstract

**Objective:**

Determine prenatal and neonatal factors that predict infantile outcomes in patients with congenital heart disease (CHD).

**Study Design:**

Retrospective cohort of 415 neonates with CHD admitted to a neonatal intensive care unit (NICU). Statistical tests included Chi-square, Fisher’s Exact, Kruskal-Wallis, and multivariable logistic regression.

**Results:**

Cardiac lesion type was associated with mortality, length of stay, and enteral feeding tube support at discharge (EFTD) (*p* ≤ 0.01). A genetic diagnosis and an extra-cardiac congenital anomaly were associated with higher odds of respiratory support needs at discharge (RSND) [OR 2.8 (95% CI: 1.2, 6.5); 4.8 (1.9, 11.8)] and EFTD [5.5 (2.9, 10.8); 3.4 (2.4–9.7)]. Lower birth weight was associated with higher odds of RSND [0.5 (0.38, 0.66)], and lower gestational age with higher odds of EFTD [0.84 (0.75, 0.95)].

**Conclusion:**

Several factors predicted adverse outcomes in infants with CHD, helping to identify high-risk cases for targeted care and improved parental guidance.

## Introduction

Congenital heart disease (CHD) is the most common birth defect and often requires surgical correction in infancy [1]. Aside from the specific type of heart defect, several additional factors impact neonatal management, including the need for immediate admission to a neonatal intensive care unit (NICU). Studies have shown that the odds of preterm birth for neonates with CHD are about 2–3 times higher than the general population risk of ~10%, and prematurity is associated with a six-fold increased risk of mortality in the six months after cardiac surgery [2–6]. Furthermore, prematurity introduces several additional neonatal morbidities including bronchopulmonary dysplasia, retinopathy of prematurity, and necrotizing enterocolitis [2]. Patients with CHD exhibit early developmental delay with up to 45% requiring feeding tubes (nasogastric or gastrostomy) to safely discharge home after undergoing cardiac surgery [7].

Additionally, the presence of an underlying genetic diagnosis predicts worse outcomes in infants with CHD. One study found that among infants who underwent CHD surgery as neonates, having a genetic diagnosis was associated with prematurity, extra-cardiac anomalies, lower weight at surgery, and a higher operative mortality rate (9.6% in patients with a genetic abnormality vs. 4.1% in patients without an identified genetic abnormality) [8]. Additionally, for patients without extra-cardiac anomalies, a genetic abnormality was independently associated with increased mortality [8]. Furthermore, the presence of genetic syndromes and/or additional anomalies are independent risk factors for lower mental development scores and overall neurodevelopmental impairment in infants with CHD [9]. Other factors that might influence neonatal and infant outcomes for patients with CHD, such as maternal age, maternal body mass index (BMI), maternal diabetes, mode of delivery, APGAR scores, and early growth have not been adequately investigated but may also contribute to adverse outcomes.

Moreover, the overall prenatal detection rate of CHD is highly variable depending on the setting of care and type of lesion. One study showed that the prenatal detection rate for critical CHD across many countries was about 50.8% with the highest detection for hypoplastic left heart syndrome at around 87.1% and the lowest detection rate for transposition of the great arteries at 40.5% and total anomalous pulmonary venous connection at 28.3% [10]. Early detection of critical CHD during pregnancy allows for more detailed assessment for additional anomalies, diagnostic genetic testing via amniocentesis or chorionic villus sampling, and referral to obstetric and pediatric specialists during the pregnancy to optimize pregnancy and early neonatal outcomes.

Currently, while there is some information known about the overall outcomes of patients with CHD and various factors that may impact neonatal outcomes, no standardized approach exists for providing a nuanced risk profile for infant mortality and overall healthcare needs during infancy for this patient population. This approach would allow clinicians to identify high-risk patients and apply targeted interventions to improve their outcomes, along with providing families with a personalized risk assessment for their child. Previous literature suggests that prenatal detection and diagnosis of CHD improves outcomes for neonates postnatally and parents desire information about expected clinical care and outcomes during the prenatal period [11–16].

Thus, the objective of this study was to determine which factors, and their associated magnitudes, were important for predicting neonatal and infant outcomes for newborns requiring admission to a NICU due to the presence of CHD. Our overarching hypothesis posits that multiple variables influence postnatal outcomes and identifying and aggregating these factors prenatally or shortly after birth will allow for early intervention, personalized treatments, and comprehensive parental counseling on expected outcomes.

## Methods

A retrospective cohort study enrolled all neonatal patients with CHD admitted to the Riley Children’s Hospital (Riley) Level IV NICU, neonatal cardiovascular intensive care unit (ICU), and/or step-down cardiac unit (Riley Heart Center) between January 2021 and December 2023 (Indiana University IRB #19046, exempt status; study performed in accordance with the Declaration of Helsinki). Exclusion criteria included patients diagnosed with CHD admitted after one month of age. During the study period, patients with atrial septal defect, ventricular septal defect, and tetralogy of Fallot without cyanosis were not routinely admitted to an ICU unless there were other indications for admission, such as preterm birth, and therefore these types of CHD were less likely to be represented in this study. Additionally, patients were excluded if they had a congenital arrhythmia or electrophysiologic abnormalities without any structural abnormalities.

At Riley, there is a NICU Nest Family Support Program, which consists of team members who are parents of NICU graduates and provide support and resources to families who are currently undergoing high-risk pregnancies or have infants with critical conditions in the ICU. In order to perform patient/family-engaged research, the study team met with NICU Nest members to elucidate which outcomes parents of children with CHD most want to know about prenatally and/or soon after NICU admission. Through those conversations, the outcomes that were of the greatest interest to parents were early survival, length of stay (LOS) of the initial hospital admission, and the need for long term breathing and feeding tube support. Based on these outcomes of interest, we sought to comprehensively interrogate all maternal, fetal, and neonatal factors that might contribute to these outcomes.

A complete list of all patients admitted to the NICU and neonatal cardiovascular ICU during the study period was sourced from multiple Riley patient registries. The electronic medical record was the primary data source for this study. Specific encounters for care sought and reviewed were initial hospitalization, subsequent hospitalizations, surgical interventions during their first year of life at Riley, and respiratory and feeding tube support. Demographic data, pregnancy information for the patient and mother, all NICU diagnoses, LOS, and infant mortality data were collected. Prenatal maternal and fetal diagnoses, fetal echo findings, prenatal testing (imaging, biochemical, and genetic), postnatal diagnoses, and postnatal echo findings were also recorded.

Furthermore, genetic testing results were collected, and the presence of additional extra-cardiac major congenital anomalies were determined with reference to the National Birth Defects Prevention Network Congenital Malformations Surveillance Report, which is reported in collaboration with the CDC and NIH [17, 18]. Only patients who were determined to have a major congenital anomaly (excluding any genetic condition) were included as having an extra-cardiac anomaly. This included disorders such as spina bifida, cleft lip/palate, intestinal atresia, abdominal wall defects and more. During the time of the study, the clinical policy of the Riley Children’s Hospital (Riley) Level IV NICU, neonatal cardiovascular ICU, and step-down cardiac unit (Riley Heart Center) was that every patient admitted with CHD received a medical genetics consult and genetic counseling unless genetic testing/counseling was done and confirmed with amniocentesis prenatally. Genetic testing available to patients over the study period included karyotype, microarray, CHD multi-gene panel, and trio exome/genome sequencing with testing determined by the geneticist providing consultation.

### Cardiac lesion classification

While there are multiple published cardiac classification systems, including the Botto classification system and the Society of Thoracic Surgeons classification system [19, 20], these systems tend to focus more on etiological, anatomical/structural differences, and/or surgical outcomes. Given that many of the outcomes examined in this study were associated with the physiologic nature of cardiac lesions, a different grouping of cardiac disease based on physiologic manifestation was used for this study. This system was developed in conjunction with neonatology and pediatric cardiology specialists based on the cardiac lesion’s ventricle status, ductal dependence, and oxygen delivery/cyanosis. The classification system was based on a five-level categorical rating: Category 1–Cyanotic heart disease with single-ventricle physiology and ductal-dependence (ex. Hypoplastic left heart syndrome, tricuspid atresia, pulmonary atresia/severe pulmonary stenosis, double-inlet left ventricle with pulmonary atresia/stenosis, unbalanced atrioventricular canal defect with pulmonary atresia/stenosis); 2–Cyanotic heart disease with two-ventricle physiology and ductal-dependent circulation (ex. tetralogy of Fallot with pulmonary atresia/stenosis, d-transposed great arteries, double-outlet right ventricle with pulmonary atresia); 3– Cyanotic heart disease with two-ventricle physiology, but not ductal-dependent (ex. tetralogy of Fallot with mild pulmonary stenosis, double-outlet right ventricle with mild pulmonary stenosis, total anomalous pulmonary venous return, truncus arteriosus); 4– Acyanotic heart disease, ductal-dependent (ex. hypoplastic left heart syndrome, hypoplastic left heart syndrome variants {such as double-outlet right ventricle, mitral stenosis/atresia, coarctation of the aorta}, pulmonary stenosis with well-balanced systemic to pulmonary circulation, interrupted aortic arch); 5–Acyanotic heart disease, not ductal-dependent (ex. Ventricular septal defect, atrioventricular septal defect, aortic stenosis, and vascular ring).

### Primary outcomes classification

The primary outcomes assessed in this study included mortality prior to first hospital discharge, LOS, respiratory support at discharge, and enteral feeding tube support at discharge. Mortality was defined as death occurring prior to discharge from the hospital, excluding patients who were discharged home on hospice care (*n* = 7) and infants transferred to another hospital prior to discharge (*n* = 4). LOS was defined as the total duration of hospitalization, including time spent at referring facilities prior to transfer to Riley for the initial hospitalization. For LOS analysis, patients who were transferred to another hospital prior to discharge were excluded. All patients who were discharged from Riley, survivors and non-survivors, were included in LOS, so LOS was calculated based on day of discharge or day of death depending on survival status. Respiratory support at discharge was classified based on the presence of either nasal cannula or tracheostomy +/- with oxygen and/or ventilator support as documented in the discharge summary at the time of discharge from Riley. Enteral feeding tube support at discharge was defined as the presence of either an orogastric/nasogastric tube, a gastrostomy tube, or a gastrojejunostomy tube, also as recorded in the Riley discharge summary.

### Statistical analyses

Analyses compared distributions of infant outcomes across various demographic and clinical variables. Categorical data are presented as proportions (%). Group comparisons utilized chi-squared (X^2^) or Fisher’s exact tests to assess differences across categorical variables. Numeric/continuous data are described using means with standard deviations (SD) and medians with interquartile ranges (IQR), as appropriate. Descriptive results were used to identify variables included in prediction models in later steps. Two-sided *p*-values with a type 1 error threshold of α < 0.05 for statistical significance were used, in addition to review of odds ratios with 95% confidence intervals. All analyses were performed in SAS version 9.4 (SAS Institute, Cary, NC, USA).

### Multivariable modeling

Two multivariable logistic regression models were developed to classify patients’ risk of requiring either respiratory support at discharge or feeding support at discharge (which were dichotomous variables, i.e., requiring vs. not requiring support). We developed both models based on a combination of causal-framework directed acyclic graphs depicting relationships between variables determined from prior clinical knowledge and results of bivariable associations identified in descriptive analyses. Several variables were associated with or mediated the outcome of requiring respiratory/feeding support. A limited set of variables were included for adjustment to reduce bias, which included the following: presence of genetic diagnoses, birth weight (treated continuously), gestational age, presence of major extra-cardiac congenital anomalies, intubation status (pre-operative or operative), CHD type (single-ventricle vs. double-ventricle physiology), need for extracorporeal membrane oxygenation, and cardiac surgery at first admission. We tested interactions, but none were significant and thus are not presented here. We summarized the final models’ output in the form of odds ratio plots and presented prediction probability plots as possible clinical aids. We confirmed the assumption of linearity between the logit and numeric variables.

### Model performance

We assessed model discrimination via area under the curve (AUC) of the receiver-operator curve and with calibration plots with 95% confidence bands. For the respiratory support model, we determined that inclusion or exclusion of the cardiac surgery at admission variable did not improve model performance via receiver-operator curve comparisons with a Mann-Whitney test of AUC differences. We used the bootstrap method for internal validation of both models, as recommended over alternative methods like split-sample or cross-validation and endorsed in TRIPOD guidelines [21, 22]. For both models, we generated 500 new datasets using random-sampling-with-replacement, compiling AUC metrics for each, and we estimated the models’ optimism scores when compared to the original models’ AUC. Optimism-corrected AUC values are provided.

## Results

### Cohort description

Patient and prenatal characteristics of the study cohort are described in Table 1A, B, respectively, and further stratified by three outcomes of interest: mortality, LOS, and enteral feeding support at hospital discharge. There were 272 infants (66%) diagnosed with CHD prenatally and 143 (34%) patients presented with symptomatic CHD shortly after birth. There were 202 infants (49%) who were inborn. The mean gestational age was 37 weeks (SD ± 2.6). The average birth weight was 3041 grams (g) (SD ± 604). Mothers with hypertension had babies with significantly lower birth weights than those without hypertension (mean= 2 907 ± 650 g vs. 3 069 ± 588 g; *p* < 0.01), as did mothers with pre-eclampsia (mean= 2 731 ± 878 g vs. 3 074 ± 562 g; *p* < 0.01). The mean maternal age at delivery was 30 years (SD ± 6). There were 39 (9%) mothers who reported tobacco use during pregnancy, 36 (9%) who reported marijuana use, and 21 (5%) who reported other illicit drug use during pregnancy. Mothers with tobacco use during pregnancy had babies with significantly lower birth weights than those without tobacco use (mean= 2820 ± 557 g vs. 3 067 ± 598 g; *p* = 0.049), as did mothers with marijuana use (mean= 2844 ± 641 g vs. 3064 ± 592 g; *p* = 0.01). There was no significant difference in birth weights for mothers with illicit drug use (mean= 2637 ± 599 g vs. 3061 ± 594 g; *p* = 0.18). A total of 389 patients (94%) had any type of genetic testing, and 85% had exome sequencing (ES)/genomic sequencing (GS). There were 118 patients (28% of the cohort) found to have a genetic diagnosis causative of the CHD phenotype, of which 78 patients (66%) had one or more copy number variants (aneuploidy or micro deletion/duplication), 36 patients (31%) had one or more single gene variants, and 4 patients (3%) had both a copy number and single gene variant. Of the 415-patient cohort, 296 infants (71%) underwent cardiac surgery during the ICU admission, 76% of survivors and 57% of non-survivors, and 51 infants (12%) required extracorporeal membrane oxygenation, 7% of survivors and 46% of non-survivors. A complete list of cardiac lesions, associated anomalies, receipt of genetic services and a diagnosis are provided in supplementary table 1.

**Table 1:** A Demographic and Patient Factors Predicting Differences in Mortality, LOS and Enteral Feeding Tube Support at Hospital Discharge. B. Maternal and Pregnancy Factors Predicting Differences in Mortality, LOS and Enteral Feeding Support at Hospital Discharge.

A	Total 415١ N (%)	Mortality at Hospital Discharge N (%)١ Total 61 (15%)٢	*P*-value	LOS of Hospital Stay Median (IQR)	*P*-value	Enteral Feeding Tube at Hospital Discharge٤ N (%)١ Total 181 (53%)	*P*-value
Sex							
Male	231 (56)	25 (11)	**0.01**	37 (55)	0.67	102 (51)	
Female	184 (44)	36 (20)		35 (58)		79 (56)	0.35
Race							
Asian	8 (2)	0 (0)		36 (30)		5 (63)	
Black or African American	66 (16)	11 (17)	0.35	39 (119)	0.60	29 (53)	0.23
White	331 (80)	47 (14)		35 (50)		146 (53)	
Other	6 (1)	2 (33)		39 (22)		0 (0)	
Ethnicity							
Hispanic	51 (12)	4 (8)	0.12	35 (37)	0.70	22 (48)	0.46
Not Hispanic	359 (87)	54 (15)		37 (57)		159 (54)	
Gestational age							
<30 weeks	15 (4)	0 (0)		124 (228)		10 (71)	
30-33 weeks	22 (5)	8 (36)	**0.02**	67 (109)	< **0.01**	11 (79)	< **0.01**
34-36 weeks	73 (18)	11 (15)		51 (61)		41 (70)	
37+ weeks	305 (73)	42 (14)		31 (40)		119 (46)	
Birth weight							
<1000 g	11 (3)	0 (0)		250 (233)		8 (80)	
1000–2000 g	45 (11)	11 (24)		82 (91)		26 (79)	
2000–3000 g	170 (41)	28 (16)	0.08	36 (50)	< **0.01**	70 (51)	< **0.01**
3000–4000 g	168 (40)	21 (13)		31 (36)		67 (47)	
>4000 g	21 (5)	1 (5)		24 (30)		10 (50)	
CHD classification٣							
1	114 (27)	32 (28)		43 (46)		49 (65)	
2	113 (27)	13 (12)		40 (52)		50 (50)	
3	50 (12)	6 (12)	< **0.01**	29 (53)	< **0.01**	18 (42)	**0.01**
4	51 (12)	2 (4)		36 (81)		46 (60)	
5	87 (21)	8 (9)		26 (31)		18 (38)	
Non-cardiac additional major congenital anomalies							
Yes	84 (20)	20 (24)	**0.01**	60 (104)	< **0.01**	47 (81)	< **0.01**
No	331 (80)	41 (13)		33 (47)		134 (47)	
Genetic diagnosis causative of CHD٥							
Yes	118 (28)	27 (23)	**0.01**	51 (83)	< **0.01**	69 (79)	< **0.01**
No	271 (65)	32 (12)		35 (45)		108 (46)	

B	Total 415١ N (%)	Mortality at Hospital Discharge N (%)١ Total 61 (15%)٢	*P*-value	LOS of Hospital Stay Median (IQR)	*P*-value	Enteral Feeding Tube at Hospital Discharge٤ N (%)١ Total 181 (53%)	*P*-value
Maternal age							
<20 years	19 (5)	2 (11)		26 (50)		6 (35)	
20-29 years	187 (45)	27 (15)	0.95	36 (50)	0.60	74 (47)	0.15
30-39 years	182 (44)	27 (15)		40 (76)		87 (58)	
40+ years	27 (7)	5 (19)		36 (76)		14 (64)	
Maternal Diabetes							
Yes	63 (15)	8 (13)	0.65	42 (48)	0.14	33 (62)	0.15
No	347 (84)	56 (15)		35 (58)		147 (51)	
Gestational or chronic hypertension							
Yes	77 (19)	10 (13)	0.61	43 (73)	**0.04**	41 (64)	**0.05**
No	333 (80)	51 (16)		35 (50)		139 (51)	
Pre-eclampsia							
Yes	41 (10)	4 (10)	0.34	66 (106)	< **0.01**	23 (64)	0.17
No	369 (89)	57 (16)		35 (50)		157 (52)	
Polyhydramnios							
Yes	21 (5)	7 (33)	**0.02**	39 (104)	**0.02**	9 (69)	0.23
No	389 (94)	54 (14)		35 (51)		171 (53)	
Intrauterine growth restriction							
Yes	60 (14)	11 (19)	0.40	43 (102)	**0.02**	32 (68)	**0.03**
No	350 (84)	50 (14)		35 (51)		148 (51)	

١Percentages may not total 100 due to missing values. Significant *p*-values are bolded.

٢4 patients with missing data for mortality at discharge due to being transferred to another hospital prior to discharge.

٣CHD Classification system:

1. Cyanotic heart disease with single-ventricle physiology.

2. Cyanotic heart disease with two-ventricle physiology and ductal-dependent circulation.

3. Cyanotic heart disease with two-ventricle physiology, but not ductal-dependent.

4. Acyanotic heart disease, ductal-dependent.

5. Acyanotic heart disease, not ductal-dependent.

٤Enteral feeding tube at discharge was only assessed in patients who survived to ICU discharge without transfer to another hospital.

٥Of patients who had genetic testing sent.

### Mortality

Within the study cohort, 61 (15%) patients died during their first ICU admission. Patient sex assigned at birth, gestational age, birth weight, type of CHD, the presence of extra-cardiac major anomalies, a genetic diagnosis, and polyhydramnios were all significantly associated with mortality. To understand why female patients had a higher mortality rate than males, 36 (20%) vs. 25 (11%); *p* = 0.01, multiple bivariate analyses were performed. In deceased patients, there were no significant associations between sex and genetic diagnosis (59 (27%) males vs. 59 (33%) females; *p* = 0.17), the presence of extra-cardiac anomalies (44 (19%) males vs. 40 (22%) females; *p* = 0.50), or cardiac lesion type (category 1 – 67 (29%) males vs. 47 (26%) females, category 2 – 61 (26%) males vs. 52 (28%) females, category 3 – 28 (12%) males vs. 22 (12%) females, category 4 – 42 (18%) males vs. 45 (24%) females, category 5 – 33 (14%) males vs. 18 (10%) females; *p* = 0.38). Given the significant association of polyhydramnios and death, multivariate analysis was also performed, and there was no significant association between polyhydramnios and presence of genetic condition (*p* = 0.21) or extra-cardiac congenital anomaly (*p* = 0.33).

### Length of hospitalization stay

The median LOS for patients who survived to discharge was 36 days (IQR 55) vs. 38 days (IQR 78) for non-survivors. Multiple factors predicted longer hospital LOS (Table 1A, B). While maternal gestational/chronic hypertension and pre-eclampsia were significantly associated with longer LOSs in univariate analysis, this was due to preterm births resulting in lower birth weights and gestational ages of patients born to mothers with these conditions.

### Respiratory support needs at discharge

There were 343 infants (83%) that survived to ICU/hospital discharge without comfort care or hospice, of which 39 (11%) had RSND (Table 2A, B). For patients that survived the ICU stay, 144 (42%) required mechanical ventilation prior to cardiac repair due to respiratory failure. For the 68 patients that did not survive the ICU admission or were discharged home on comfort care, 61 (90%) required mechanical ventilation prior to any cardiac procedure due to respiratory failure. While cardiac lesion type was significantly associated with RSND (*p* = 0.02) in univariate analysis, it was not after multivariable adjustment, accounting for intubation status prior to surgical repair, presence of extra-cardiac major congenital anomalies, and presence of genetic diagnoses (*p* = 0.19). Similarly, race was significantly associated with RSND in univariate analysis, however, it was not significantly associated after multivariable adjustment due to gestational age differences that existed between racial groups. Black/African American infants were more likely to be born preterm with 15% of infants born less than 34 weeks gestation compared to 6% of White infants born less than 34 weeks (*p* = 0.04). Similarly, there was a significant association between race and birth weight with Black/African American infants having an average birth weight of 2845 ± 583 g and White infants with an average birth weight of 3094 ± 583 g (*p* = 0.02).

**Table 2:** A. Demographic and Patient Factors Predicting Differences in Respiratory Support at Hospital Discharge. B. Maternal and Pregnancy Factors Predicting Differences in Respiratory Support at Hospital Discharge.

A Total 343١ N (%)	None *N* = 304 (89)٢	*P*-value	Cannula Support *N* = 23 (7)٢	*P*-value	Tracheostomy Support *N* = 16 (5)٢	*P*-value
Gender						
Male	181 (90)	0.40	12 (6)	0.51	8 (4)	0.47
Female	123 (87)		11 (8)		8 (6)	
Race						
Asian	8 (100)		0 (0)		0 (0)	
Black or African American	41 (75)	< **0.01**	5 (9)	0.71	8 (15)	**0.02**
White	248 (91)		18 (7)		8 (3)	
Other	4 (100)		0 (0)		0 (0)	
Ethnicity						
Hispanic	41 (89)	0.85	2 (4)	0.49	3 (7)	0.46
Not Hispanic	261 (88)		21 (7)		13 (4)	
Gestational age						
<30 weeks	9 (64)		3 (21)		2 (14)	
30-33 weeks	8 57)	< **0.01**	3 (21)	< **0.01**	3 (21)	< **0.01**
34-36 weeks	47 (80)		6 (10)		6 (10)	
37+ weeks	238 (93)		11 (4)		5 (2)	
Birth weight						
<1000 g	4 (40)		3 (30)		3 (30)	
1000–2000 g	20 (61)	< **0.01**	8 (24)	< **0.01**	5 (15)	< **0.01**
2000–3000 g	124 (91)		8 (6)		5 (4)	
3000–4000 g	136 (94)		4 (3)		3 (2)	
>4000 g	20 (100)		0 (0)		0 (0)	
CHD classification٣						
1	70 (92)		5 (7)		1 (1)	
2	93 (93)	**0.02**	5 (5)	0.65	2 (2)	**0.01**
3	38 (88)		2 (5)		3 (7)	
4	44 (92)		3 (6)		1 (2)	
5	59 (77)		8 (10)		9 (12)	
Non-cardiac additional major congenital anomalies٤						
Yes	38 (66)	< **0.01**	13 (22)	< **0.01**	7 (12)	< **0.01**
No	266 (93)		10 (3)		9 (3)	
Genetic diagnosis causative of CHD٤						
Yes	66 (76)	< **0.01**	12 (14)	**0.02**	9 (10)	< **0.01**
No	214 (92)		11 (5)		7 (3)	

B Total 343١ N (%)	None *N* = 304 (89)٢	*P*-value	Cannula Support *N* = 23 (7)٢	*P*-value	Tracheostomy Support *N* = 16 (5)٢	*P*-value
Maternal age						
<20 years	14 (82)		1 (6)		1 (6)	
20-29 years	139 (89)	0.51	12 (8)	0.86	5 (3)	0.63
30-39 years	133 (89)		8 (5)		8 (5)	
40+ years	19 (82)		2 (9)		2 (9)	
Maternal Diabetes						
Yes	47 (89)	0.91	1 (2)	0.12	5 (9)	0.08
No	252 (88)		22 (8)		11 (4)	
Gestational or chronic hypertension						
Yes	50 (78)	**0.01**	7 (11)	0.14	7 (11)	**0.01**
No	249 (91)		16 (6)		9 (3)	
Pre-eclampsia						
Yes	27 (75)	**0.02**	6 (17)	**0.01**	3 (8)	0.28
No	272 (90)		16 (6)		13 (4)	
Polyhydramnios						
Yes	12 (92)	0.64	0 (0)	0.32	1 (8)	0.47
No	287 (88)		23 (7)		15 (5)	
Intrauterine growth restriction						
Yes	33 (70)	< **0.01**	8 (17)	**0.01**	6 (13)	**0.01**
No	266 (91)		15 (5)		10 (3)	

١Percentages may not total 100 due to missing values. Significant *p*-values are bolded.

٢4 patients with missing data for mortality at discharge due to being transferred to another hospital prior to discharge.

٣CHD Classification system:

1. Cyanotic heart disease with single-ventricle physiology.

2. Cyanotic heart disease with two-ventricle physiology and ductal-dependent circulation.

3. Cyanotic heart disease with two-ventricle physiology, but not ductal-dependent.

4. Acyanotic heart disease, ductal-dependent.

5. Acyanotic heart disease, not ductal-dependent.

٤Of patients who had genetic testing sent.

**Fig. 1:**
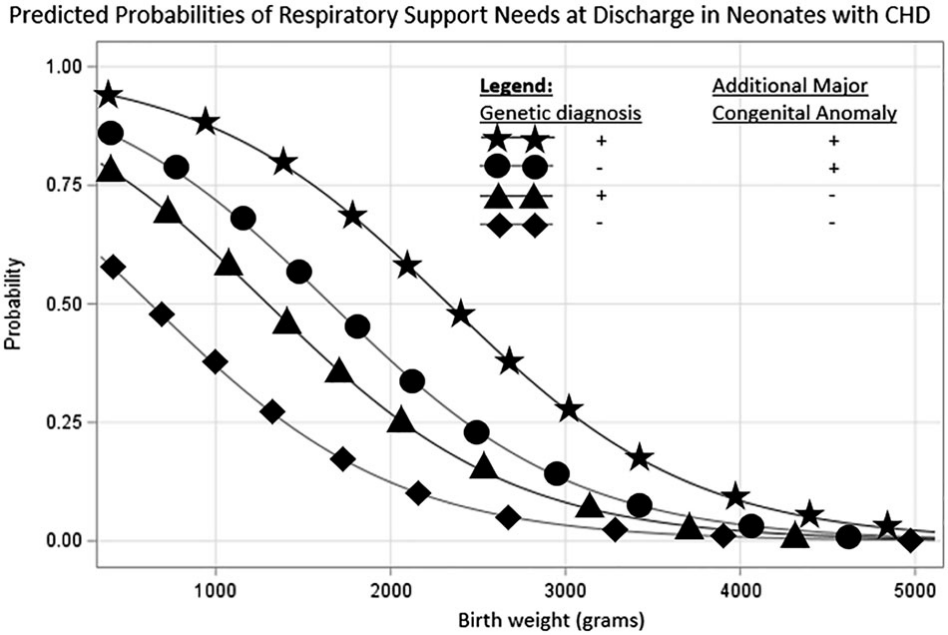
Predicted probabilities of respiratory support at hospital discharge based on birth weight, presence of genetic diagnosis causative of CHD, and presence of additional extra-cardiac major congenital anomalies in neonates with symptomatic CHD.

Table 3 describes variables that were significantly associated with RSND. While larger birth weights and receiving cardiac surgery during the first ICU admission were protective against RSND, an underlying genetic diagnosis causative of CHD, presence of extra-cardiac major congenital anomaly, and need for intubation prior to cardiac surgery all resulted in higher odds of RSND. Using the variables of birth weight, presence of genetic diagnoses, and presence of extra-cardiac major congenital anomalies, the prediction model for RSND (Fig. 1) had good discriminative performance [AUC = 0.86 (95% CI: 0.79, 0.92)] (Table 3). When adjusting the model for cardiac surgery during the first admission and intubation status prior to cardiac surgery, model performance improved further [AUC = 0.89 (0.82, 0.95)], though this was not statistically significant (*p* = 0.10). Based on internal validation, the optimism-corrected AUC was 0.87. This indicates that intubation status, presence of other major congenital anomalies, birth weight, and presence of genetic diagnoses were most predictive of RSND.

**Table 3:** Variables Associated with Respiratory Support Needs at Hospital Discharge in Neonates with CHD.

_Variable_	_β Coefficient [Standard Error]_	_Wald X_^2^_(*p*-value)_	_Odds Ratio [95% CI]_	_Area Under the Curve [95% CI]_
_Birth weight_	_-.001 [0.0003]_	_24.85 (_ < _0.01)_	_0.50 [0.38, 0.66]_	_0.89 [0.82, 0.95]_
_Non-cardiac additional major congenital anomaly_	_1.56 [0.46]_	_11.48 (_ < _0.01)_	_4.78 [1.93, 11.82]_	
_Intubated prior to cardiac procedure_	_2.43 [0.93]_	_6.79 (0.01)_	_11.30 [1.82, 70.01]_	
_Genetic diagnosis causative of CHD_	_1.02 [0.44]_	_5.36 (0.02)_	_2.77 [1.17, 6.54]_	
_Cardiac surgery within the first ICU admission_	_-0.25 [0.58]_	_0.18 (0.67)_	_0.78 [0.25, 2.46]_	

### Enteral feeding support needs at discharge

Of the 343 patients who survived to ICU/hospital discharge without comfort care or hospice, 181 infants (53%) required an enteral feeding tube at discharge from the ICU. Furthermore, 247 infants (72%) required fortification of enteral feeds at discharge. When performing multivariable analysis, variables/factors displayed in Table 4 were found to be significantly associated with enteral feeding tube support at discharge. Cardiac lesion type was significantly associated with feeding support at discharge when conducting the univariate analysis (*p* < 0.01), and having a genetic diagnosis was associated with higher odds of feeding support [OR 5.5 (95% CI: 2.9, 10.8)] at discharge, as was having an extra-cardiac major congenital anomaly [3.4 (1.6, 7.5)]. Using the variables of gestational age, intubation prior to cardiac intervention, presence of genetic diagnoses, presence of extra-cardiac major congenital anomalies, single vs. double-ventricle physiology of the cardiac lesion, and need for extracorporeal membrane oxygenation during the initial hospitalization, the prediction model for enteral feeding support at discharge (Table 4) had acceptable discriminative performance [AUC = 0.80 (0.75, 0.85)]. The optimism-corrected AUC was 0.79.

**Table 4:** Variables Associated with Enteral Feeding Tube Support Needs at Hospital Discharge in Neonates with CHD.

_Variable_	_β Coefficient [Standard Error]_	_Wald X_^2^_(*p*-value)_	_Odds Ratio [95% CI]_	_Area Under the Curve [95% CI]_
_Genetic diagnosis causative of CHD_	_0.86 [0.17]_	_25.39 (_ < _0.01)_	_5.54 [2.85, 10.79]_	_0.80 [0.75, 0.85]_
_Intubated prior to cardiac procedure_	_0.88 [0.20]_	_20.04 (_ < _0.01)_	_4.83 [2.19, 10.63]_	
_Non-cardiac additional major congenital anomaly_	_0.61 [0.20]_	_9.32 (_ < _0.01)_	_3.41 [1.55, 7.48]_	
_Ventricle status (single ventricle)_	_0.49 [0.17]_	_8.47 (_ < _0.01)_	_2.67 [1.38, 5.17]_	
_Gestational age_	_-0.17 [0.06]_	_7.42 (_ < _0.01)_	_0.84 [0.75, 0.96]_	
_Need for ECMO during ICU hospitalization_	_1.05 [0.39]_	_7.08 (_ < _0.01)_	_8.17 [1.74, 38.38]_	

## Discussion

This large, single-center cohort study identified key outcomes for newborns requiring ICU care due to CHD and the factors that most significantly influenced those outcomes. A comprehensive approach was taken to interrogate the relevance of all knowable prenatal and early neonatal factors that might influence the outcomes of mortality, LOS, RSND, and feeding support at hospital discharge.

Many studies have shown a link between preterm births and CHD with some studies indicating that an infant with CHD has a 2–3 times likelihood of delivering preterm [4]. Our cohort similarly had a preterm birth rate of 27%, higher than the national preterm birth rate of 10% [23]. However, two potentially modifiable prenatal complications, prenatal drug exposure and maternal pre-eclampsia, in part influenced parturition and birth weight in our cohort. While gestational age and birth weight are established drivers of neonatal mortality and morbidity, the reasons for these complications differed by patient demographic in our cohort. For example, Black/African American infants had significantly lower birth weights and gestational ages compared to their White counterparts, likely in part due to higher rates of pre-eclampsia (21% vs. 8%, *p* < 0.01). Furthermore, rates of hypertensive disorders were higher in our cohort overall than what is reported in a national cohort of pregnant women, ~15 vs. 5–7%, respectively [24, 25]. This is not surprising given that hypertensive disorders in pregnancy are associated with congenital heart defects in offspring [26]. Moreover, even when hypertensive disorders of pregnancy were further subdivided into gestational hypertension, chronic hypertension and pre-eclampsia, there was still a significantly increased risk of CHD in offspring [26].

Unfortunately, our study indicated that newborns with symptomatic CHD suffered significant early mortality with 15% of patients dying during their initial hospital admission. Female sex, moderate preterm gestational age, very low birth weight category, single-ventricle CHD lesions, presence of additional extra-cardiac major anomalies, and having a primary genetic diagnosis were all associated with worse mortality. The disparity in mortality experienced by female patients has not been previously described. This finding contrasts sharply with typical NICU outcomes, where females generally exhibit better survival rates [27]. Furthermore, a review of previous studies has shown evidence that males are typically born with more severe CHD, and females tend to have milder cardiac lesions [28], however there was no difference in lesion types within our cohort. This disparity indicates an important area for future investigation to identify underlying factors contributing to differential outcomes based on sex.

Our study further confirms that the presence of an underlying genetic diagnosis predicts worse outcomes in infants with CHD, as seen in previous studies [8]. Our study showed that having an underlying genetic condition and/or extra-cardiac anomaly increased the odds of needing respiratory support or enteral feeding support at hospitalization discharge. Previous studies have shown that the presence of genetic syndromes/anomalies is associated with lower mental development scores and overall neurodevelopmental impairment in infants with CHD, which was not evaluated in this study, but an area of future investigation [9]. LOS varied significantly based on the type of cardiac lesion, indicating that physiological categorization of CHD may facilitate more targeted counseling in the future to give more precise estimates of LOS prenatally based on fetal echocardiogram findings.

Hospital course analyses and disposition of survivors revealed important findings. Birth weight demonstrated a logarithmic relationship with respiratory support needs; notably, neonates weighing below 2000 g faced markedly increased risks, especially if additional extra-cardiac anomalies or genetic diagnoses were present. This illustrates the importance of birth weight for neonates with CHD and the significance of prenatal development prior to the infant being delivered. Furthermore, gestational age as opposed to birth weight was found to be a predictor for enteral feeding support needs at the time of discharge. This aligns with the thought that oral-motor skills required for feeding are a developmental milestone, and therefore gestational age rather than birth weight is a more appropriate metric [29]. Additionally, as previous studies have shown, premature infants typically need more calories to gain weight and grow compared to term infants, and this is likely to be exacerbated by the addition of a cardiac lesion, making it even more challenging for preterm infants with CHD discharged from an ICU to go home without enteral feeding support [30, 31].

In considering study limitations, this cohort analysis was from a single center, which limits generalizability and does not account for practice variation across centers. Additionally, outcomes were described for the initial neonatal ICU admission, and longer-term infant or early childhood outcomes were not evaluated. Findings from this study provide a foundation for future studies with larger sample sizes of patients with CHD to ultimately create a more accurate estimate of expected outcomes in similar populations, and those exploring the long-term implications of this approach on neonatal outcomes.

Using the novel approach outlined in the current study to determine prenatal and early neonatal factors that predict infantile outcomes in patients with CHD has significant implications for both clinicians and families. For example, having such information will allow clinicians to identify infants with CHD that may be at a higher risk for medical complications and help inform what targeted treatments they should receive to optimize their outcomes whilst in the NICU. Additionally, using such an approach may better prepare families regarding the medical needs and range of outcomes for their child, leading to improved psychological outcomes for parents/caregivers and health outcomes for their child.

## Conclusions

To summarize, this study examined factors affecting key outcomes for hospitalized newborns with CHD using a novel, functional cardiac lesion grouping system. Our examination of prenatal, hospital, and post-discharge outcomes identified key predictive factors associated with early mortality, prolonged hospitalization, and developmental delay. Overall, post-discharge outcomes highlight ongoing concerns in neonates with CHD, as a notable proportion of infants experienced developmental delays, specifically respiratory and feeding difficulties, and required continued home healthcare support. These findings reinforce the critical nature of early identification, multidisciplinary management, and holistic care approaches beginning prenatally and extending beyond the NICU stay.

## Supplementary information

Table S1

## Data Availability

De-identified data is available from the corresponding author upon reasonable request and with the appropriate agreements in place.
