# Supplementary material for: Predicting the future for neonates with symptomatic congenital heart disease
Source: J Perinatol. 2026 Apr 13;46(7):1146–54. doi: 10.1038/s41372-026-02653-6 (PMC13423830; doi:10.1038/s41372-026-02653-6)
Supplement: Supplementary file 1 — Table S1 [file 41372_2026_2653_MOESM1_ESM.docx]

| **Table S1.** Complete list of cardiac lesions, associated anomalies, receipt of genetic services and diagnoses | | | | | |
| --- | --- | --- | --- | --- | --- |
| **Cardiac class** | **Cardiac diagnosis** | **Extra-cardiac anomalies** | **Inpatient genetic consult** | **Genetic testing** | **Genetic diagnosis that explains the CHD** |
| 1 | Unbalanced AV canal, coarctation | None | Yes | Yes | Trisomy 21 - CNV |
| 1 | DILV, severe left AV valve stenosis and pulmonary atresia, Single ventricle with left AV valve atresia, LTGA, pulmonary atresia | None | Yes | Yes | 1q21.1q21.2 deletion (1.29 Mb) - CNV |
| 1 | Pulmonary atresia with intact IVS, tricuspid stenosis, RV hypoplasia | None | Yes | Yes | Noonan syndrome (PTPN11) - SNV |
| 1 | DILV, hypoplasic RV (functional single ventricle), straddling TV, VSD | micrognathia, cleft palate | Yes | Yes | CHARGE syndrome (CHD7) - SNV |
| 1 | Severe Ebstein's anomaly, moderate to severe TR, near PA | None | Yes | Yes | 16q24.2q24.3 deletion - CNV |
| 1 | Pulmonary atresia w/ intact ventricular septum, moderately hypoplastic RV | None | Yes | Yes | 15q25.1q25.2 deletion syndrome - CNV |
| 1 | Coarc, PDA,unbalanced AV canal | duodenal atresia, imperforate anus | Yes | Yes | Trisomy 21 - CNV |
| 1 | unbalanced AV canal defect with small LV, interrupted arch, consistent with HLHS | diaphragmatic eventration | Yes | Yes | CHARGE syndrome (CHD7) - SNV |
| 1 | complete AV canal unbalanced, coarctation | None | No | Yes | Trisomy 21 - CNV |
| 1 | DORV, subaortic VSD, hypoplastic left heart syndrome with near PA | None | Yes | Yes | SNIP1-related disorder & atypical distal 22q11.2 deletion (does not include HIRA or TBX1) - CNV and SNV |
| 1 | Truncus Arteriosus type 1, large secundum ASD, large anterior malaligned VSD, moderate transverse aortic arch hypoplasia, severe discrete coarctation | None | Yes | Yes | GATA6 - SNV |
| 1 | tricuspid atresia, severe coarctation | imperforate anus, cleft palate | Yes | Yes | Trisomy 22 - CNV |
| 1 | severe shone's complex (significant coarct), pulm stenosis, tricuspid stenosis, severe aortic stenosis | None | Yes | Yes | FLNA-related disorder - SNV |
| 1 | Unbalanced AVC with DORV, D-TGA, HLV, severe PS with MAPCAS | Agenesis of corpus callosum, cleft lip/palate, heterotaxy | Yes | Yes | CDH2-related disorder - SNV |
| 1 | Hypoplastic left heart syndrome. moderate hypoplastic ascending/transverse arch, moderate coarct | None | Yes | Yes | Turner syndrome - CNV |
| 1 | Mitral Atresia, Hypoplastic LV, DORV with side by side great vessels, moderate to large ASD | None | Yes | Yes | 8p23.1 duplication, 19p13.3 deletion - CNV |
| 1 | DORV w/ subaortic VSD, mild aortic verriding, malignment VSD, severe hypoplastic mitral valve w/ severe stenosis | Phocomelia of bilateral upper extremities, syndactyly | Yes | Yes | TBX5-related disorder (Holt-Oram syndrome) - SNV |
| 1 | Shone's Complex, complete AV canal defect, mildly inbalanced, mild hypoplastic LV, hypoplastic and trileaflet Ao valve, tunnel subaortic stenosis, Ao arch hypoplasia | None | Yes | Yes | 4q deletion syndrome - CNV |
| 1 | HLHS | None | Yes | Yes | SIN3A-related disorder (Witevee-Kolk syndrome) - SNV |
| 1 | HLHS (MA/AA), small ASD | unilateral renal agenesis | Yes | Yes | Kabuki syndrome (KMT2D) - SNV |
| 1 | HLHS, truncus arteriosus | None | Yes | Yes | Kleefstra syndrome (9q34 microdeletion syndrome) - CNV |
| 1 | Hypoplastic RV, pulmonary atresia, near tricuspid atresia | None | Yes | Yes | No |
| 1 | Ebstein's anomaly and functional pulmonary atresia | None | Yes | Yes | No |
| 1 | HLHS with mitral stenosis and aortic atresia, small ASD, abnormal dilation of L coronary system | None | Yes | Yes | No |
| 1 | DORV, HLH, Coarctation, supracardiac TAPVR | left renal agenesis | Yes | Yes | No |
| 1 | HRV, DILV, TGA, severely hypoplastic aorta and abnormal pulmonary valve | None | Yes | Yes | No |
| 1 | HLHS, intact atrial septum | None | Yes | Yes | No |
| 1 | HLHS, severe coarctation of the aorta | None | Yes | Yes | No |
| 1 | HRV w/ severely hypoplastic TV, PDA, mod PS, mod ASD, small VSD | None | Yes | No | Unknown |
| 1 | Pulmonary atresia with instact septum, hypoplastic RV | None | Yes | Yes | No |
| 1 | HRHS, pulmonary valve atresia, BAV | None | Yes | Yes | No |
| 1 | Hypoplastic RV, pulmonary atresia with intact ventricular septum | None | Yes | No | Unknown |
| 1 | Hypoplastic right heart, pulmonary stenosis, tricuspid atresia | None | Yes | Yes | No |
| 1 | Hypoplastic right heart, pulmonary atresia | imperforate anus | Yes | Yes | No |
| 1 | Severe ebstein's anomaly | None | Yes | Yes | No |
| 1 | pulmonary atresia with intact ventricular septum, tricuspid valve atresia, severely hypoplastic RV | None | Yes | Yes | No |
| 1 | hypoplastic RV, dextrocardia | None | Yes | Yes | No |
| 1 | tricuspid atresia, d-TGA, hypoplastic transverse aorta, discrete coarct | None | Yes | Yes | No |
| 1 | tricuspid atresia, hypoplastic RV | None | Yes | Yes | No |
| 1 | tricuspid atresia, hypoplastic RV, interrupted aortic arch, transposition of great arteries | esophageal atresia with fistula, imperforate anus | No | Yes | No |
| 1 | severe hypoplastic RV, severe tricuspid stenosis, pulmonary atresia with intact septum | None | Yes | Yes | No |
| 1 | Severe Ebstein's anomaly (severe apical displacement of tricuspid valve), RVOT | None | Yes | Yes | No |
| 1 | severe ebstein's anomaly | None | Yes | Yes | No |
| 1 | tricuspid atresia, hypoplastic RV w/ subvalvar outflow obstruction, R aortic arch | None | Yes | Yes | No |
| 1 | hypoplastic RV, RVH, pulmonary insufficiency, tricuspid insufficiency | None | Yes | Yes | No |
| 1 | pulmonary atresia, tricuspid stenosis, hypoplastic right heart | None | Yes | Yes | No |
| 1 | Ebsteins anomaly, pulmonary atresia, moderate to large secundum ASD | None | Yes | Yes | No |
| 1 | tricuspid atresia, pulmonary stenosis | None | Yes | Yes | No |
| 1 | severe tricuspid valve regurgitation, pulmonary valve atresia with retrograde flow in ductus arteriosus, cardiomegaly | None | Yes | Yes | No |
| 1 | unbalanced AV canal, TAPVR, MAPCAs | imperforate anus, renal anomalies | Yes | Yes | No |
| 1 | Unbalanced AV canal, pulmonary atresia, TAPVR, heterotaxy | None | Yes | Yes | No |
| 1 | pulmonary valve atresia, tricuspid atresia, severe R ventricular hypoplasia | None | Yes | Yes | No |
| 1 | tricuspic atresia, hypoplastic RV, VSD, no RVOT | None | No | Yes | No |
| 1 | DILV, tricuspid atresia, D-TGA, coarctation | None | Yes | Yes | No |
| 1 | Hypoplastic right heart with severe pulmonary stenosis | None | Yes | Yes | No |
| 1 | critical pulmonary stenosis, HRV | None | Yes | Yes | No |
| 1 | tricuspid atresia, hypoplastic RV, pulmonary atresia, VSD with overriding aorta bilateral SVCs | None | Yes | Yes | No |
| 1 | hypoplastic RV, pulmonary atresia, severe TS | None | Yes | Yes | No |
| 1 | severe pulmonary valve stenosis, ebsteins anomaly | None | Yes | Yes | No |
| 1 | Dextrocardia, unbalanced AV canal, PAPVR, DORV, pulmonary atresia | Heterotaxy | Yes | Yes | No |
| 1 | HRHS, pulm atresia, intact ventricular septum, hypoplastic tricuspid valve, hypoplastic RV | None | Yes | Yes | No |
| 1 | Severe Ebstein's anomaly, moderate to severe TR, mild to mod pulm regurg | None | Yes | Yes | No |
| 1 | DILV, L-TGA, hypoplastic PA | None | Yes | Yes | No |
| 1 | Critical pulmonary stenosis, severely hypoplastic TV/RV | None | Yes | Yes | No |
| 1 | Severe pulmonary atresia, hypoplastic RV | None | No | No | Unknown |
| 1 | dextrocardia, complex single ventricle anatomy, d-TGA, pulmonary atresia, L AV valve atresia | unilateral duplex kidney | Yes | Yes | No |
| 1 | Pulmonary atresia, severely stenotic tricuspid valve, moderately hypoplastic RV | None | Yes | Yes | No |
| 1 | Severe ebstein's anomaly, devere displacement of posterior leaflet to near RV apexr, moderate to severe tricuspid insufficiency | None | Yes | Yes | No |
| 1 | DORV, complete AV canal defect moderately unbalanced, pulmonary stenosis | unilateral choanal atresia, cleft palate, abnormal genitalia | Yes | Yes | No |
| 1 | Coarctation of aorta, Right dominant AV canal, AVSD | biliateral Club feet, Short limbs, facial dysmorphism | No | No | Unknown |
| 1 | HLHS | None | Yes | Yes | No |
| 1 | DORV, d-TGA, BAV, subpulmonic VSD, severe coarct | None | Yes | Yes | No |
| 1 | HLHS variant (severe mitral and aortic stenosis), hypoplastic aortic valve | None | Yes | Yes | No |
| 1 | hypoplastic left heart syndrome, coarct | jejunal atresia | Yes | Yes | No |
| 1 | hypoplastic left heart syndrome | None | Yes | Yes | No |
| 1 | Hypoplastic left heart syndrome | cleft lip | Yes | Yes | No |
| 1 | Hypoplastic left heart syndrome, coarctation | None | Yes | No | Unknown |
| 1 | HLHS, DORV, Coarctation / hypoplastic ascending aorta | None | Yes | Yes | No |
| 1 | HLHS | None | Yes | No | Unknown |
| 1 | DILV with L AV valve stenosis, normally related unobstructed great arteries off single ventricle, and hypoplastic arch | None | Yes | Yes | No |
| 1 | Shone's complex, DORV, subaortic VSD, coarct | None | Yes | Yes | No |
| 1 | hypoplastic left heart syndrome (mitral atresia/aortic atresia) | None | Yes | Yes | No |
| 1 | Dextrocardia, HRV, DORV, TA, ASD, VSA, Coarctation of aorta | None | Yes | Yes | No |
| 1 | Hypoplastic left heart syndrome | None | Yes | yes | No |
| 1 | Unbalanced AV canal with mild-mod AV valve regurgitation | None | Yes | Yes | No |
| 1 | DILV, DORV, severely hypoplastic left AVV, heterotaxy with L atrial isomerism, coarctation of aorta | None | Yes | Yes | No |
| 1 | Severely unblanaced AV canal, heterotaxy, D-TGA and pulm atresua | Heterotaxy, asplenia | Yes | Yes | No |
| 1 | HLHS (MS/AA), aneurysmal atrial septum with a small superiorly located atrial level communiction | None | Yes | Yes | No |
| 1 | HLHS | None | Yes | Yes | No |
| 1 | HLHS | None | Yes | Yes | No |
| 1 | HLHS with aortic and mitral atresia | None | Yes | Yes | No |
| 1 | HLHS | None | Yes | No | Unknown |
| 1 | HLHS, mildly dilated RV, mild TR | None | Yes | Yes | No |
| 1 | HLHS, aitral and aortic valve atresia, pulmonary vein stenosis | heterotaxy | Yes | Yes | No |
| 1 | HLHS | None | Yes | Yes | No |
| 1 | HLHS | None | Yes | Yes | No |
| 1 | HLHS | None | Yes | Yes | No |
| 1 | DILV, d-TGA, severe coarctation, hypoplastic RV | None | Yes | Yes | No |
| 1 | HLHS (MA/AA), small ascending arch | None | Yes | Yes | No |
| 1 | HLHS (MA/AA), small ascending arch | None | Yes | Yes | No |
| 1 | TGA/DORV, complex AV canal w/ atretic mitral component/hypoplastic LV, single ventricle, Heterotaxy | Heterotaxy | Yes | Yes | No |
| 1 | HLHS (MS/AA), small ASD | None | Yes | Yes | No |
| 1 | HLHS (MA/AA), small ASD, ascending and transverse arch severely hypoplastic arch | None | Yes | Yes | No |
| 1 | HLHS (MS/AA), small ASD | None | Yes | Yes | No |
| 1 | Unbalanced AV canal defect, moderately hypoplastic LV, d-TGA | Heterotaxy, cleft lip/palate | Yes | Yes | No |
| 1 | HLHS (MS/AS), severe LV hypoplasia, hypoplastic ascending aorta and arch | None | Yes | Yes | No |
| 1 | HLHS (MA/AA) | None | Yes | Yes | No |
| 1 | HLHS (MA/AA) - hypoplastic LA, mod dilated RA/RV, hypoplastic ascending aorta | None | Yes | No | Unknown |
| 1 | Dextrocardia, unbalanced R dominant AV canal, severely hypoplastic L ventricle, moderately dilated R ventricle, pulmonary valve atresia | None | Yes | Yes | No |
| 1 | HLHS - severely hypoplastic left ventricle, severely hypoplastic aortic and mitral valves | None | Yes | Yes | No |
| 1 | severe pulmonary stenosis | None | Yes | Yes | No |
| 1 | severe pulmonary stenosis | None | Yes | Yes | No |
| 1 | severe pulmonary valve stenosis | None | Yes | Yes | No |
| 1 | Hypoplastic right heart, tricuspid atresia, severely hypoplastic RV | None | Yes | No | Unknown |
|  |  |  |  |  |  |
| 2 | VSD, overriding aorta, hypoplastic PV, significantly hypoplastic branched PAs, secundum ASD | omphalocele | Yes | Yes | 22q11.2 deletion syndrome - CNV |
| 2 | Tetraology of fallot, severe pumonary stenosis | TEF, cleft lip/palate | Yes | Yes | Unbalanced translocation with large duplication in chormosome 3 and deletion in chromosome 11 - CNV |
| 2 | Critical pulmonary valve stenosis | unilateral club foot | Yes | Yes | Saethre-Chotzen Syndrome (TWIST1 gene deletion) - SNV |
| 2 | Tetraology of Fallot with pulmonary atresia and MAPCAs | unilateral renal agenesis | Yes | Yes | 22q11.2 deletion syndrome - CNV |
| 2 | TOF with pulmonary atresia, confluent PAs | None | Yes | Yes | SNIP1-related disorder - SNV |
| 2 | L-TGA, large VSD, severe left ventricular outflow tract obstruction, pulmonary atresia | None | Yes | Yes | NODAL gene deletion - CNV |
| 2 | Tetrology of Fallot, near pulmonary atresia | None | Yes | Yes | FLT4-related disorder - CNV |
| 2 | d-TGV, small VSD | None | Yes | Yes | TRRAP-related disorder - SNV |
| 2 | TOF with absent pulmonary valve | None | Yes | Yes | 22q11.2 deletion syndrome - CNV |
| 2 | DORV, TGA, dextrocardia | situs inversus, heterotaxy | Yes | Yes | FLT4-related disorder - SNV |
| 2 | Pulmonary atresia, large anterior malaignment VSD, multiple MAPCAs | None | Yes | Yes | TBX1-related disorder - SNV |
| 2 | ToF w/ absent pulmonary valve, severe subvalvular stenosis, severe pulm insufficency | None | Yes | Yes | 22q11.2 deletion syndrome - CNV |
| 2 | ToF - large malaignment VSD, borderline hypoplastic pulmonary arteries, RVOT narrowing | Hirschsprung's disease | No | Yes | Trisomy 21 - CNV |
| 2 | ToF w/ severe PS, large anterior malaignment VSD | cutis aplasia, retinal detachment | Yes | Yes | Adams-Oliver syndrome (DOCK6) - SNV |
| 2 | ToF w/ severe PS, MAPCAs | None | Yes | Yes | FLT4-related disorder - SNV |
| 2 | DORV/TOF, AV canal defect, moderate dynamic RVOT/subpulmonary stenosis, large inlet VSD | None | Yes | Yes | T21 - CNV |
| 2 | Severe pulmonary stenosis | None | Yes | Yes | 2 diagnoses: COLA4A1 and NF1 - both SNV |
| 2 | complex TGV, d-TGA, VSD | cleft palate, hypogonadism, upper limb anomaly, unilateral optic nerve coloboma, congenital absence of unilateral ulna | Yes | Yes | CHARGE syndrome (CHD7) - SNV |
| 2 | D-TGA vs DORV with non-committed VSD, pulmonary atresia, VSD, MAPCAs | cleft palate | Yes | Yes | Trisomy 13 (13q12q34 copy number gain) - CNV |
| 2 | interrupted aortic arch, balanced AV canal defect | None | No | Yes | Trisomy 21 - CNV |
| 2 | Shone's complex (coarctation and severely hypoplastic transverse aortic arch) | cleft palate, hirschsprung's disease | Yes | Yes | Unbalanced chromosomal translocation (18q22.1q23 del, 20p13p11.23 dup) - CNV |
| 2 | coarctation of aorta, hypoplastic LV | None | Yes | Yes | Monosomy X (mosaic) - CNV |
| 2 | balanced AV canal, coarctation of the aorta | None | No | Yes | Trisomy 21 - CNV |
| 2 | interrupted aortic arch, VSD | None | Yes | Yes | Trisomy 21 - CNV |
| 2 | non-compaction cardiomyopathy hypoplastic transverse arch, moderate coarctation | None | Yes | Yes | 1p36 deletion syndrome - CNV |
| 2 | Complete AVCD, Small Ao arch and coarctation | Cleft lip, Cleft palate, unilateral renal agenesis | Yes | Yes | CHARGE sequence (CHD7) - SNV |
| 2 | DORV, sub-aortic VSD, IAA | R multicystic kidney, severe unilateral hydronephrosis, bilateral club feet, Dandy Walker, ventriculomegaly | Yes | Yes | unbalanced translocation (partial trisomy with large duplication of chromosome 5 and small deletion of chromosome 13) - CNV |
| 2 | IAA (type B), VSD, small aortic valve | None | Yes | Yes | 22q11.2 deletion syndrome - CNV |
| 2 | Interrupted Ao arch, VSD | None | Yes | Yes | 22q11.2 deletion syndrome - CNV |
| 2 | VSD, IAA type | None | Yes | Yes | 22q11.2 deletion syndrome - CNV |
| 2 | IAA, VSD, ASD | None | Yes | Yes | 22q11.2 deletion syndrome - CNV |
| 2 | Shone's complex | None | Yes | Yes | Noonan's syndrome (PTPN11) - SNV |
| 2 | DORV, dTGA, AVC, PS, Heterotaxy | heterotaxy, asplenia | Yes | Yes | No |
| 2 | dTGA, mod muscular VSD | None | Yes | Yes | No |
| 2 | DORV, DTGA, VSD, coarctation | None | Yes | Yes | No |
| 2 | Hypopastic transverse arch with severe coarctation, large perimembranous VSD | cleft lip, cleft palate | Yes | Yes | No |
| 2 | TOF with PA and VSD | None | Yes | Yes | No |
| 2 | DORV with subpulmonic VSD, pulmonary atresia | None | Yes | Yes | No |
| 2 | d-TGA with intact ventricular septum, restrictive ASD | None | Yes | Yes | No |
| 2 | severe pulmonary stenosis | None | Yes | Yes | No |
| 2 | d-TGA | None | Yes | Yes | No |
| 2 | d-TGA with intact atrial septum | None | Yes | Yes | No |
| 2 | DORV, d-TGA, pulm stenosis, LSVC | TEF | Yes | Yes | No |
| 2 | DORV with PA, balanced AV canal | None | Yes | Yes | No |
| 2 | tetraology of fallot with pulmonary atresia | None | Yes | Yes | No |
| 2 | d-TGA | Microtia | Yes | Yes | No |
| 2 | tetraology of fallot with pulmonary atresia | myelomeningocele | Yes | Yes | No |
| 2 | DORV with subpulmonic VSD, d-TGA mild pulmonary stenosis, restrictive PFO | None | Yes | Yes | No |
| 2 | DORV, pulmonary stenosis | Omphalocele | Yes | Yes | No |
| 2 | transposition of great vessels, DORV, PA | None | Yes | Yes | No |
| 2 | tetraology of fallot, severe RVOT | None | Yes | Yes | No |
| 2 | transposition of great vessels, intact ventricular septum, restrictive ASD | None | Yes | Yes | No |
| 2 | tetraology of fallot, pulmonary atresia | Dandy Walker | Yes | Yes | No |
| 2 | dTGA, small secundum ASD, Large perimembranous VSD | None | Yes | No | Unknown |
| 2 | tetraology of fallot with severe RV outflow obstruction | None | Yes | Yes | No |
| 2 | dTGA with nearly intact atrial septum | None | Yes | Yes | No |
| 2 | DORV, dTGA, subpulmonic VSD | None | Yes | Yes | No |
| 2 | TOF with pulmonary atresia and hypoplastic confluent branched pulmonary artiers with secundum ASD | None | Yes | Yes | No |
| 2 | d-TGA with intact ventricular septum and restrictive ASD | None | Yes | Yes | No |
| 2 | d-TGA, coarct, subpulmonary VSD, Taussig Bing anatomy | None | Yes | Yes | No |
| 2 | balanced AV canal, DORV, d-TGA, supracardiac TAPRV, R aortic arch, severe PS, Heterotaxy | Heterotaxy | Yes | Yes | No |
| 2 | D-TGA with intact ventricular septum and restrictive atrial septum | None | Yes | Yes | No |
| 2 | dTGA, single coronary artery, small to moderate perimembranous VSD | None | Yes | Yes | No |
| 2 | dTGA | None | Yes | Yes | No |
| 2 | Critical pulmonic stenosis | None | Yes | Yes | No |
| 2 | DORV with L-malposed great arteries, inlet VSD, subpulmonary and pulmonary stenosis, common AV canal | Heterotaxy | Yes | Yes | No |
| 2 | TOF, severe pulmonary stenosis, large VSD | None | Yes | Yes | No |
| 2 | VSD, dextrocardia, pulmonary atresia | diaphragmatic hernia | Yes | No | Unknown |
| 2 | d-TGA w/ intact ventricular septum | None | Yes | Yes | No |
| 2 | d-TGA, small to mod VSD, thickened pulm valve leaflets | None | Yes | Yes | No |
| 2 | dTGA | None | Yes | Yes | No |
| 2 | dTG with moderate but unrestrictive VSD | None | Yes | Yes | No |
| 2 | DORV, DTGA, VSD | None | Yes | No | Unknown |
| 2 | DORV, dTGA, hypoplastic pulmonary valve | None | Yes | Yes | No |
| 2 | DORV, VSD, dTGA, PS | heterotaxy | Yes | Yes | No |
| 2 | DORV with sub-aortic VSD and coarctation of aorta | None | Yes | Yes | No |
| 2 | DTGA, subpulmonic valve narrowing, VSD, ASD | None | Yes | Yes | No |
| 2 | d-TGA, pulm atresia, VSD | None | Yes | Yes | No |
| 2 | d-TGA w/ intact septum | None | Yes | Yes | No |
| 2 | ToF w/ PA, RVOT obstruction, large VSD | None | Yes | No | Unknown |
| 2 | ToF w/ PA, RVOT obstruction, large VSD | None | Yes | Yes | No |
| 2 | d-TGA w/ small ASD | None | Yes | Yes | No |
| 2 | L-TGA, pulmonary valve atresia, hypoplastic pulmonary arteries | None | Yes | No | Unknown |
| 2 | d-TGA w/ small ASD | None | Yes | No | Unknown |
| 2 | d-TGA, small ASD | None | Yes | No | Unknown |
| 2 | d-TGA, small VSD, slight malaignment of aortic and pulmonary valve commisures | None | Yes | Yes | No |
| 2 | DORV w/ uncommitted VSD, no LVOT obstruction, severe RVOT obstruction, moderately hypoplastic PA annulus, severe pulmonary valve stenosis | None | Yes | Yes | No |
| 2 | d-TGA, VSD, ASD | None | Yes | Yes | No |
| 2 | d-TGA, coarctation of aorta | None | Yes | Yes | No |
| 2 | d-TGA w/ mod ASD | None | Yes | No | Unknown |
| 2 | Critical pulmonary valve stenosis, moderate RVH, tricuspid regurgitation | None | Yes | Yes | No |
| 2 | DORV w/ subaortic VSD, moderately hypoplastic mtrial valve | None | Yes | Yes | No |
| 2 | d-TGA w/ small ASD | None | Yes | Yes | No |
| 2 | d-TGA w/ small ASD | None | Yes | Yes | No |
| 2 | d-TGA w/ small ASD | None | Yes | Yes | No |
| 2 | d-TGA | None | Yes | yes | No |
| 2 | d-TGA w/ restrictive ASD | None | Yes | Yes | No |
| 2 | DORV/ToF, malaignment VSD, mild PS | None | Yes | Yes | No |
| 2 | ToF w/ mild turned severe PS | unilateral renal agensis | Yes | Yes | No |
| 2 | heterotaxy, L-looped cardiac chambers, DORV w non-committed VSD and side-by-side arteries, pulmonary stenosis, complete mildly unblanaced AV cana defect | Heterotaxy | Yes | Yes | No |
| 2 | d-TGV, VSD, straddling tricuspid valve, large ASD | None | Yes | Yes | No |
| 2 | d-TGA w/ VSD | None | Yes | Yes | No |
| 2 | Shone's complex (hypoplastic aortic arch, severe coarctation of the aorta) | None | Yes | Yes | No |
| 2 | critical aortic stenosis, LVH | None | Yes | Yes | No |
| 2 | interrupted aortic arch | None | Yes | Yes | No |
| 2 | d-TGA, VSD, coarct | None | Yes | Yes | No |
| 2 | severe coarctation of aorta | None | Yes | Yes | No |
| 2 | BAV, Moderate VSD, mildly hypoplasic aortic arch | None | Yes | Yes | No |
| 2 | critical aortic stenosis, mitral stenosis | None | Yes | Yes | No |
| 2 | Hypoplastic transverse aortic arch with discrete coarctation, large posterior malaigned VSD, dilated PA with PV regurg | None | Yes | Yes | No |
| 2 | IAA | None | Yes | Yes | No |
| 2 | Critical aortic stenosis, dilated LV w severely decreased LV function | None | Yes | Yes | No |
| 2 | d-TGA w/ restrictive ASD | None | Yes | Yes | No |
|  |  |  |  |  |  |
| 3 | DORV with subaortic VSD, mild PS | None | Yes | Yes | Trisomy 18 - CNV |
| 3 | DORV, VSD | None | Yes | Yes | 12p13.33p13.32 deletion, 12q24.33 deletion - CNV |
| 3 | large VSD, DORV with subaortic VSD | omphalocele | Yes | Yes | Trisomy 18 - CNV |
| 3 | TAPVR (type 1) | None | Yes | Yes | 17p13.3 microduplication syndrome - CNV |
| 3 | DORV, subaortic VSD | diaphragmatic hernia | Yes | Yes | ABL1-related disorder - SNV |
| 3 | DORV, subaortic VSD, side-to-side arteries, mod PS, R aortic arch | None | Yes | Yes | 22q11.21 deletion - CNV |
| 3 | Truncus arteriosus | absent thymus, unilateral clubfoot | Yes | Yes | 22q11.2 deletion syndrome - CNV |
| 3 | tetraology of fallot, mild RVOT | imperforate anus, vertebrae anomalies | Yes | Yes | UPB1 variant - SNV |
| 3 | DORV with VSD, mild PS | None | Yes | Yes | Trisomy 21 - CNV |
| 3 | ToF, mild pulmonary stenosis | None | Yes | Yes | 1q21.1 duplication syndrome - CNV |
| 3 | TOF with compete AV canal | None | Yes | Yes | Trisomy 21 - CNV |
| 3 | Truncus arteriosus type 2, tricuspid atresia | None | Yes | Yes | GATA6-related disorder - SNV |
| 3 | TAPVR, infracardiac with mod obstruction, mod ASD, mod severely dilated RV | None | Yes | Yes | 1q21.1 deletion syndrome - CNV |
| 3 | Type 1 truncus arteriosus, small PFO vs secundum ASD, hypovolemic LV, large anterior malalignment VSD | None | yes | Yes | No |
| 3 | Hemi-truncus and VSD (RPA arising from ascending aorta) | None | Yes | Yes | No |
| 3 | DORV with subaortic VSD, mild pulm stenosis, hypoplasia of RVOT | None | Yes | Yes | No |
| 3 | d-TGA with intact ventricular septum | None | Yes | Yes | No |
| 3 | truncus arteriosus | None | Yes | Yes | No |
| 3 | Tetraology of Fallot with mild sub pulmonic stenosis | None | Yes | Yes | No |
| 3 | Type 1 truncus arteriosis with overridle large VSD, moderate ASD, small coarc | None | Yes | Yes | No |
| 3 | DORV, subaortic VSD, mild PS | None | Yes | Yes | No |
| 3 | truncus arteriosus | None | Yes | Yes | No |
| 3 | tetraology of fallot, mild RVOT, vascular ring | None | Yes | Yes | No |
| 3 | truncus arteriosus | None | Yes | Yes | No |
| 3 | Unobstructed TAPVR | None | Yes | Yes | No |
| 3 | DORV with subaortic VSD, partial ectopia cordis | omphalocele | Yes | Yes | No |
| 3 | DORV, VSD | None | Yes | Yes | No |
| 3 | TAPVR | None | Yes | Yes | No |
| 3 | TOF with mild pulmonary stenosis | unilateral multicystic dysplastic kidney | Yes | Yes | No |
| 3 | TOF, mild pulm stenosis | None | Yes | Yes | No |
| 3 | ToF, mild supravalvular and valvular stenosis, no pulmonary valve insufficiency | None | No | Yes | No |
| 3 | ToF, low normal pulmonary valve annulus, small to mod PDA | None | Yes | Yes | No |
| 3 | ToF, perimembranous VSD, aortic override, moderate PS | None | Yes | No | Unknown |
| 3 | DORV, subaortic VSD, no outflow tract obstructions | None | Yes | Yes | No |
| 3 | ToF, doming pulmonary valve with no significant gradient | None | Yes | No | Unknown |
| 3 | DORV w/ subaortic VSD, aortia posterior to PA | None | Yes | Yes | No |
| 3 | ToF, mild pulmonary stenosis | None | No | Yes | No |
| 3 | Supracardiac TAPVR | None | Yes | Yes | No |
| 3 | DORV with side by side great vessels and moderate PS | None | Yes | Yes | No |
| 3 | Unbalanced LV dominant AV canal, DORV with sub pulmonary VSD and pulmonary stenosis | None | Yes | Yes | No |
| 3 | TAPVR | hypospadias | Yes | Yes | No |
| 3 | TOF, mild PS | None | Yes | Yes | No |
| 3 | d-TGA, VSD, ASD, mod PS | None | Yes | Yes | No |
| 3 | DORV w/ PS, unbalanced complete common AV canal, TAPVR | None | Yes | Yes | No |
| 3 | Truncus arteriosus type 1, bicuspic truncal valve, truncal valve stenosis | None | Yes | Yes | No |
| 3 | ToF, mild pulmonary stenosis, significant sub-valvular component | None | Yes | Yes | No |
| 3 | Truncus, large VSD | None | Yes | Yes | No |
| 3 | Intracardiac TAPVR to coronary sinuses, moderate ASD | None | Yes | Yes | No |
| 3 | ToF, moderate RVH, long segment subpulmonary valve narrowing with tunnel-like RVOT | unilateral dysplastic kidney | Yes | Yes | No |
| 3 | Non-obstructed infradiaphragmatic TAPVR | None | Yes | Yes | No |
|  |  |  |  |  |  |
| 4 | coarctation of the aorta, HCM w/ dynamic LVOT obstruction, mild pulmonary stenosis, mod-large ASD | None | Yes | Yes | Noonan syndrome (PTPN11) - SNV |
| 4 | balanced AV canal, severe coarctation | None | Yes | Yes | Trisomy 21 - CNV |
| 4 | severe coarctation, mildly hypoplastic transverse aortic arch | None | Yes | Yes | Trisomy 21 - CNV |
| 4 | coarctation of aorta, hypoplastic transverse aortic arch, no right subclavian vein, right inominate vein | pulmonary hypoplasia, polycystic kidney disease | Yes | Yes | 2 diagnoses: 22q11.2 duplication syndrome & PEX1-related peroxisomal biogenesis disorder & PKHD1-related polycystic kidney disease - CNV and SNV |
| 4 | Shone complex, Moderate PFO vs secundum ASD, midly hypoplastic LV, aortic coarctation, high muscular VSD | Hypospadias | Yes | Yes | 5q31-q35 duplication - CNV |
| 4 | Shones complex (BAV, mildly hypoplastic LV, coarctation, VSD) | cystic kidneys | Yes | Yes | 2 diagnoses: Trisomy 21 and ADPKD - CNV and SNV |
| 4 | Shone's Complex and coarctation of Ao, BAV | None | Yes | Yes | Turner syndrome - CNV |
| 4 | Coarctation, balanced AV canal | duodenal atresia | Yes | Yes | Trisomy 21 - CNV |
| 4 | Severe Coarctation of aorta | None | Yes | Yes | ABCC9-related disorder (Cantu syndrome) - SNV |
| 4 | Shone's Complex - coarct, hammock type mitral valve, hypoplasia of proximal arch | None | Yes | Yes | Kabuki syndrome (KMT2D) - SNV |
| 4 | Shone's complex, hypoplastic transverse arch/coarct, mild MV stenpsis and mod-severe stenotic AV | None | Yes | Yes | No |
| 4 | Shones Complex | None | Yes | Yes | No |
| 4 | Severe coarctation, BAV | None | Yes | Yes | No |
| 4 | severe coarct, severe transverse arch hypoplasia, posterior malaignment VSD | None | Yes | Yes | No |
| 4 | severe coarct | cleft palate | Yes | Yes | No |
| 4 | severe coarctation of aorta | None | Yes | Yes | No |
| 4 | critical coarctation of the aorta | None | Yes | Yes | No |
| 4 | moderate coarctation of the aorta, hypoplastic arch | None | Yes | Yes | No |
| 4 | Shone's complex (severe coarct, BAV, hammock MV) | None | Yes | Yes | No |
| 4 | critical coarctation | None | Yes | Yes | No |
| 4 | severe coarctation | None | Yes | Yes | No |
| 4 | severe coarctation of aorta | None | Yes | Yes | No |
| 4 | severe coarctation of aorta | None | Yes | Yes | No |
| 4 | Critical Coarctation of Aorta | None | Yes | Yes | No |
| 4 | severe coarctation of aorta | None | Yes | Yes | No |
| 4 | severe coarctation | None | Yes | Yes | No |
| 4 | severe coarctation of aorta | None | Yes | Yes | No |
| 4 | coarctation of aorta, bicuspid aortic valve | None | Yes | Yes | No |
| 4 | moderate coarctation, VSD | None | Yes | Yes | No |
| 4 | large VSD, CoA, hypoplastic arch | None | Yes | Yes | No |
| 4 | Severe, discrete coarct, Shone's complex, abnormal mitral valve, large VSD | None | Yes | Yes | No |
| 4 | Severe coarctation | None | Yes | Yes | No |
| 4 | coarctation, hypoplastic transverse arch, large PDA, small VSD, hammock MV, BAV | None | Yes | Yes | No |
| 4 | Coarctation of aorta, right sided heart, PAPVR | imperforate anus | Yes | Yes | No |
| 4 | Parachute mitral valve, Shone's complex (coarctation), BAV | None | Yes | Yes | No |
| 4 | Moderate coarctation, large VSD, hammock mitral valve, BAV | None | Yes | Yes | No |
| 4 | Severe coarctation with distal displacement of L subclavian, BAV | None | Yes | Yes | No |
| 4 | ASD, severe coarctation | None | No | Yes | No |
| 4 | L-TGA, large VSD, possible coarct | None | Yes | Yes | No |
| 4 | Coarct, mildly hypoplastic transverse arch, large posterior malaignment VSD | None | Yes | Yes | No |
| 4 | Coarctation of aorta, abnormal mitral valve | None | Yes | Yes | No |
| 4 | Coarctation of aorta | None | Yes | Yes | No |
| 4 | Coarctation of aorta, R sided arch, aberrant L subclavian artery, vascular ring | None | Yes | Yes | No |
| 4 | Severe coarctation | None | Yes | No | Unknown |
| 4 | Severe coarctation, hypoplastic transverse arch, large posterior malaignment VSD | None | Yes | Yes | No |
| 4 | Shone's complex, Coarctation of aorta, L aortic arch, large perimembranous VSD | unilateral renal agensis, anorectal malformation | Yes | Yes | No |
| 4 | Coarctation | None | Yes | Yes | No |
| 4 | Coarctation | None | Yes | No | Unknown |
| 4 | AV canal w/ mild L-sided hypoplasia, coarctation of aorta | None | Yes | Yes | No |
| 4 | Shone's Complex - severe discrete coarct, hypoplastic arch, likely hammock mitral valve | None | Yes | No | Unknown |
| 4 | Severe Coarctation | None | Yes | No | Unknown |
|  |  |  |  |  |  |
| 5 | severe pulmonary valve stenosis with mild to mod regurg | None | Yes | Yes | Duplication 9p with unbalanced transloction between chromosomes 5 and 9 - CNV |
| 5 | balanced intermediate AV acanal | Hypospadias | No | Yes | Trisomy 21 - CNV |
| 5 | Inlet VSD, mass on TV | Dandy Walker, coloboma | Yes | Yes | Trisomy 18 - CNV |
| 5 | multiple VSDs, moderate tricuspid regurg, moderate PDA, BAV | esophogeal atresia with fistula | Yes | Yes | Trisomy 18 - CNV |
| 5 | Interrupted IVC with azygous continuation to RVSC | heterotaxy, jejunal atresia | Yes | Yes | Primary Ciliary Dyskinesia (DNAH9) - SNV |
| 5 | balanced AV canal, coarct | None | No | Yes | Trisomy 21 - CNV |
| 5 | LV non-compaction, dilated cardiomyopathy | corneal clouding/cataract | Yes | Yes | Rubinsten-Taybi syndrome (CREBBP) - SNV |
| 5 | Complete balanced AV canal | None | No | Yes | Trisomy 21 - CNV |
| 5 | moderate pulmonary stenosis | None | Yes | Yes | NF1 - SNV |
| 5 | R aortic arch with vascular ring, hypertrophic cardiomyopathy | None | Yes | Yes | Trisomy 21 - CNV |
| 5 | balanced AV canal | None | Yes | Yes | 2 diagnoses: Trisomy 21 & Klinefelter syndrome - both CNV |
| 5 | mod-severe pulmonary stenosis, VSD, ASD, PDA | cleft palate | Yes | Yes | 2 diagnoses: Trisomy X, interstitial deletion of 1q21 - both CNV |
| 5 | ASD, VSD, BAV, TV prolapse | limb deficiencies | Yes | Yes | Trisomy 18 - CNV |
| 5 | Multiple VSDs, LV noncompaction | Shortening of limbs | Yes | Yes | Beckwith-Wiedemann syndrome (unbalanced translocaton with terminal deletion of 1p and terminal duplication of 11p) - CNV |
| 5 | pulmonary vein stenosis | None | Yes | Yes | MAST1-related disorder (Mega-corpus callosum syndrome) - SNV |
| 5 | Right aortic arch, vascular ring | Omphalocele | Yes | Yes | KCNH1-related disorder - SNV |
| 5 | mild coarctation | cleft palate | Yes | Yes | 2 diagnoses: Trisomy 21 & Treacher-Collins syndrome - CNV and SNV |
| 5 | PAPVR | Omphalocele | Yes | Yes | Trisomy 21 - CNV |
| 5 | complete AV canal defect balanced | None | No | Yes | Trisomy 21 - CNV |
| 5 | complete AV canal unbalanced | None | No | Yes | Trisomy 21 - CNV |
| 5 | mildly stenotic L pulm artery, mild valvar to supravalvar pulm stenosis, mild LVH | cleft palate | Yes | Yes | Cornelia de Lange syndrome (NIPBL) - SNV |
| 5 | mild coarctation, common origin of L carotid and R innominate artery, BAV | None | Yes | Yes | 15q11.2 deletion - CNV |
| 5 | Complete balanced AV septal defect | duodenal atresia | Yes | Yes | 16q24.1 deletion: FOXF1-related disorder (Alveolar capillary dysplasia) - CNV |
| 5 | VSD, vascular ring | None | Yes | Yes | 22q11.2 deletion syndrome - CNV |
| 5 | Pulmonary vein stenosis, valve insufficiencies, septal defects (VSD), severe tricuspid | None | Yes | Yes | Trisomy 21 - CNV |
| 5 | PAPVR, supravalvular aortic stenosis with narrowing of the sinotubular junction | None | Yes | Yes | Williams syndrome (7q11.23 deletion) - CNV |
| 5 | complete balance AV canal | None | yes | Yes | Trisomy 21 - CNV |
| 5 | Complete balanced AV canal defect | cataracts | No | Yes | Trisomy 21 - CNV |
| 5 | Complete balanced AV canal defect | None | Yes | Yes | Trisomy 21 - CNV |
| 5 | Mildly unbalanced complete AV canal defect | clubfoot | No | Yes | Trisomy 21 - CNV |
| 5 | Complete AV canal defect, mildly unbalanced | None | Yes | Yes | Trisomy 21 - CNV |
| 5 | complete balanced AV canal, mild coarc, aberrant right subclavian | None | Yes | Yes | Trisomy 21 - CNV |
| 5 | Large VSD, aneurysmal interarterial septum, AV canal defect | None | Yes | Yes | 2 diagnosies: Trisomy 21 & Klinefelter syndrome - both CNV |
| 5 | ALCAPA | unilateral kidney hypoplasia | Yes | Yes | 22q11.2 deletion syndrome - CNV |
| 5 | Aorto-pulmonary window | Cleft lip/palate | Yes | Yes | CHARGE syndrome (CHD7) - SNV |
| 5 | Tricuspid regurg | None | Yes | Yes | Trisomy 21 - CNV |
| 5 | Transitional AV canal defect - mildly unbalanced with inflow commitment greater toward the right, Moderate to large primum ASD. chordal tissue within the inlet portion of the VSD, posterior shelf/coarct | None | Yes | Yes | 3q29 duplication syndrome - CNV |
| 5 | Ebstein's anomaly - mod apical deviation of tricuspid septal leaflet, mild tethering of tricuspid anterior leaflet | None | Yes | Yes | MYH7-related disorder - SNV |
| 5 | Mild coarctation, mildly hypoplastic transverse arch | None | Yes | Yes | TUBB-related disorder - SNV |
| 5 | Completely balanced AV canal defect, mild PS | None | No | Yes | Trisomy 21 - CNV |
| 5 | Complete AV Canal | Hypospadias | Yes | Yes | DONSON gene - SNV |
| 5 | Coarctation | None | Yes | Yes | 16p11.2 deletion syndrome - CNV |
| 5 | Small Aortic arch with BAV, mod coarct | None | Yes | Yes | No |
| 5 | mild-mod supravalvar pulmonary stenosis | None | No | Yes | No |
| 5 | pulmonary valve stenosis, multiple VSDs | None | Yes | Yes | No |
| 5 | vascular ring | None | Yes | Yes | No |
| 5 | severe LV non-compaction | None | Yes | Yes | No |
| 5 | mild-mod aortic stenosis | None | Yes | Yes | No |
| 5 | Valvular/supravalvular pulmonic stenosis | None | Yes | Yes | No |
| 5 | L pulmonary vein stenosis | None | Yes | Yes | No |
| 5 | VSD, mild pulmonary stenosis | None | Yes | Yes | No |
| 5 | cor triatriatum, mild tricuspid regurgitation | None | Yes | Yes | No |
| 5 | mild pulmonary stenosis | duodenal atresia | Yes | Yes | No |
| 5 | pulm vein stenosis, interrupted IVC, heterotaxy | None | Yes | Yes | No |
| 5 | LVH, LV dilation | None | Yes | Yes | No |
| 5 | balanced AV canal | cleft palate | Yes | Yes | No |
| 5 | Critical Aortic stenosis | None | Yes | Yes | No |
| 5 | moderate pulmonary stenosis | None | Yes | No | Unknown |
| 5 | Right PA atresia, VSD, PDA | None | Yes | yes | No |
| 5 | thickened pulmonary valves, multiple VSDs | None | Yes | Yes | No |
| 5 | scimitar syndrome, PAPVR | None | No | Yes | No |
| 5 | double aortic arch, vascular ring | none | Yes | Yes | No |
| 5 | tricuspid stenosis, mild pulmonary valve stenosis, large VSD | None | Yes | Yes | No |
| 5 | double aortic arch, VSD | None | Yes | Yes | No |
| 5 | pumonary vein stenosis | None | Yes | Yes | No |
| 5 | overlapping aorta causing tracheal compression | Shortening of arm | Yes | Yes | No |
| 5 | Mild pulmonary valve stenosis | None | No | Yes | No |
| 5 | LV septal hypertrophy, pulmonary artery stenosis | None | Yes | Yes | No |
| 5 | RV hypertrophy, tricuspid valve stenosis | unilateral club foot | Yes | Yes | No |
| 5 | Hypertrpohic cardiomyopathy with LVOT | None | Yes | Yes | No |
| 5 | right aortic arch, vascular ring | None | Yes | Yes | No |
| 5 | dysplastic/arcarde mitral valve with severe insufficiency, mild mitral stenosis, small PFA, severely dilated LA, dysplastic tricuspid valve with mild TR | None | Yes | Yes | No |
| 5 | Left ventricular dysfunction with dilated left atrium and pulmonary congestion | None | No | Yes | No |
| 5 | L-TGA, perimembranous VSD, moderate pulmonary stenosis | None | Yes | Yes | No |
| 5 | mild Coarctation of aorta, pulmonary artery stenosis | None | No | Yes | No |
| 5 | Severe pulmonary valve stenosis with RVH | None | Yes | Yes | No |
| 5 | Tricuspid atresia, large VSD | small dysplastic kidney, rectal atresia | Yes | Yes | No |
| 5 | Aortic stenosis, thickened bicuspid aortic valve with fusion of R and noncoronary leaflets - moderate stenosis | None | Yes | Yes | No |
| 5 | tricuspid atresia and VSD | None | Yes | Yes | No |
| 5 | Hypoplastic transverse aortic arch, mildly hypopastic mitral valve | None | Yes | Yes | No |
| 5 | Double aortic arch, vascular ring | None | Yes | Yes | No |
| 5 | Coarctation of aorta | None | No | Yes | No |
| 5 | Slightly thickened tricuspid valve leaflet tips (Ebsteinoid like) | None | No | Yes | No |
| 5 | Ebstein's anomaly - RVH, moderte tricuspic regurg, abnormal tricuspid valve, LV noncompaction | None | Yes | No | Unknown |
| 5 | Mitral and tricuspid regurgitation | None | Yes | Yes | No |
| 5 | Pulmonary atresia, VSD | None | Yes | No | Unknown |
| 5 | L-TGA w/ ventricular inversion | None | Yes | Yes | No |
